# AI self-efficacy and anxiety among university teachers

**DOI:** 10.1038/s41598-026-59141-2

**Published:** 2026-06-22

**Authors:** Houyu Wu, Eng Hoe Wee

**Affiliations:** 1 School of Artificial Intelligence, Neijiang Normal University, Hongqiao Road, Neijiang, 64100 Sichuan China; 2 Faculty of Education, Languages, Psychology and Music, SEGi University, Kota Damansara, Selangor, 47810 Malaysia

**Keywords:** Artificial intelligence, AI Self-efficacy, AI anxiety, University teachers, Technology adoption, Education, Psychology, Psychology

## Abstract

With the rapid integration of AI into higher education, teachers’ psychological responses are critical for technology adoption. This study examines AI self-efficacy and AI anxiety among university teachers in a Chinese university. It investigates the relationship between these two constructs and explores differences based on gender, age and academic major. A quantitative survey was administered to 350 teachers selected through stratified random sampling based on major. Results showed that both AI self-efficacy and AI anxiety were significantly above the neutral midpoint (M = 4.48, SD = 0.76; M = 4.35, SD = 0.85). AI self-efficacy was strongly and negatively associated with AI anxiety (*r* = − 0.59, *p* <0.01) and remained a significant negative predictor after controlling for gender, age, and major (β = − 0.112, *p* = .026). Female teachers reported higher anxiety and lower self-efficacy than male teachers, whereas computer science teachers reported the highest self-efficacy and the lowest anxiety. These findings suggest that university teachers may feel simultaneously capable of using AI and apprehensive about its broader implications. The study provides evidence from Chinese higher education and highlights the value of differentiated institutional support that addresses both teachers’ confidence in using AI and their professional concerns.

## Introduction

Artificial Intelligence (AI) has rapidly emerged as a transformative wave in education, holding promise for enhancing teaching and learning at every educational stage. In recent years, the rapid growth of AI-based educational technologies (AI EdTech), ranging from adaptive learning systems to generative AI tools like ChatGPT, has gained significant attention^1,2^. Studies suggest that AI can personalize instruction, offer real-time feedback, automate routine tasks such as automated essay scoring, and assist teachers in allocating resources more effectively^3^. For instance, recent surveys reveal that teachers acknowledge a range of AI benefits, including improved curriculum planning, better insights into student learning, the delivery of instant feedback through automated tools, and stronger assessment methods^4,5^. Furthermore, systematic reviews highlight that AI-driven support can improve students’ learning conditions by enabling more efficient planning and data-informed interventions^6^.

Despite these potential benefits, the actual classroom adoption of AI remains limited. The empirical understanding of AI’s impact on teaching and learning is still nascent with a lack of widespread application of AI EdTech^7^. This gap between the potential of AI and its practical implementation can be partly attributed to teachers’ readiness and attitudes (Hazzan-Bishara et al.^8^). Teachers are pivotal to educational innovation, and their perspectives on AI will significantly shape how these tools are integrated into pedagogical approaches^9^. The introduction of any new technology can create a sense of vulnerability, that teachers may worry it might not work as expected or could introduce new challenges in their classrooms^10^. The willingness to embrace this vulnerability depends fundamentally on trust in technology. In essence, a teacher’s decision to adopt AI involves weighing expected benefits against perceived risks^11^. Indeed, individuals are less likely to experiment with new technology when they perceive substantial risk, underscoring how anxiety and trust are key precursors to technology use^12^.

Teachers face multiple distinct challenges when considering AI adoption, spanning technical, pedagogical, and psychological dimensions. A frequently cited issue is the lack of AI literacy and training, as many teachers report unfamiliarity with how AI algorithms function or how to effectively integrate AI tools into their lesson plans^13^. This gap can diminish confidence and willingness to experiment. Furthermore, inadequate technological infrastructure and support present significant barriers. In many educational settings, schools may lack the necessary reliable AI software, robust IT support, or clear institutional guidance to implement AI effectively^14^. Even when tools are available, teachers often report insufficient technical assistance or support structures, leading to feelings of being technologically intimidated, as noted in qualitative studies^15–17^.

Beyond these concerns, teachers also grapple with anxieties regarding the potential impact of AI on their professional roles and student development. These concerns include worries that AI may alter teachers’ professional roles, including fears of job displacement and reduced classroom autonomy. For instance, anxiety specifically concerning job displacement is a key dimension of the apprehension experienced by Chinese university language teachers regarding AI^18^. Some teachers also worry that AI could render their professional skills obsolete. These anxieties align with the concept of AI anxiety, understood as a stress response to the uncertain future implications of AI for an individual’s career and skills^19^. Whereas broader technology anxiety generally refers to apprehension or resistance associated with using digital tools^20^, and technostress denotes the broader strain arising from technology-related demands such as overload, complexity, and uncertainty (Yang et al.^21^, AI anxiety in this study refers more specifically to concerns triggered by AI systems and their implications for human agency, professional roles, and decision making. Consistent with the AI Anxiety Scale adopted in this study, such anxiety encompasses concerns related to learning AI techniques, job replacement, sociotechnical blindness, and AI configuration^22^. Recent evidence further suggests that AI-related hindrance stressors operate through AI anxiety rather than through more general technostress pathways^23^, reinforcing the value of examining AI anxiety as a distinct construct in educational research. Meanwhile, anxieties concerning students also arise. For example, teachers express concerns about students misusing AI for tasks like cheating^24^. Further worries include AI negatively affecting learning processes, diminishing human oversight, or exacerbating issues of unequal access. Indeed, teachers’ anxiety about student learning outcomes, particularly with the adoption of tools like ChatGPT, is a primary factor shaping their attitudes toward AI^10^. Consequently, teachers often find themselves balancing enthusiasm for AI’s promise with significant apprehension about its potential risks.

These interconnected factors underscore the increasing scholarly attention devoted to the emerging constructs of AI self-efficacy and AI anxiety. Recent work confirms that teachers’ AI self-efficacy is a multidimensional belief structure that directly influences their willingness to experiment with AI tools^2,26^. AI self-efficacy reflects a teacher’s confidence in their capacity to effectively understand and utilize AI technologies. It is conceptually rooted in Bandura’s theory of self-efficacy, which defines self-efficacy as the belief in one’s capabilities to execute tasks. AI self-efficacy is also similar to established technology self-efficacy constructs within educational technology research^27^. A four-factor Artificial Intelligence Self-Efficacy Scale (AISES) showed that higher AISE scores significantly predicted teachers’ engagement in AI-supported instructional design, explaining 32% of the variance even after controlling for prior technology experience^26^. AI anxiety, in contrast, represents teachers’ apprehension or fear regarding AI adoption, essentially referring to the negative emotional response or stress experienced when considering AI in educational settings^28^. The explicit scholarly investigation of both constructs has emerged in recent years. For instance, the critical need to understand an individual’s perceived self-efficacy concerning the use of AI technologies has been emphasized, informing the formal definition of AI self-efficacy and the development of an AI Self Efficacy Scale^26^. Longitudinal evidence further indicates that generative-AI self-efficacy is the strongest predictor of teachers’ subsequent integration of ChatGPT into lesson preparation and student activities across a full semester, even when job demands and resources are accounted for^29^. Similarly, AI anxiety is conceptualized as a response to the expanding influence of AI, a psychological state considered suitable for measurement and warranting investigation within educational contexts^18^.

Much of the prior work on AI self-efficacy and AI anxiety has concentrated on K-12 teachers or pre-service teachers. For instance, among K-12 teachers, AI-related self-efficacy is one of two direct determinants of their intentions to learn about AI^30^. Furthermore, many studies concerning technology adoption in schools implicitly involve similar constructs of self-efficacy and anxiety. The advent of ChatGPT has also stimulated research on these constructs. Among university professors, technology-related anxiety, particularly concerns about student learning, strongly influences the ChatGPT adoption intentions of female instructors^1^. Pre-service teachers also demonstrate these dynamics, for example, generative AI anxiety predicts their intention to design AI-assisted lessons^31^. In China, the AI self-efficacy of K-12 teachers strongly predicts their intention to engage with AI learning^30^. Similarly, five dimensions of AI anxiety, including concerns about technical proficiency and job displacement, have been identified among Chinese university English teachers, highlighting the distinct challenges these educators perceive with AI^18^.

Nevertheless, AI self-efficacy and AI anxiety specifically among university teachers remain underexplored^32^. University teachers operate within a distinct professional context. They often possess more autonomy than K-12 teachers, concentrate on specialized content and research, and encounter different demands, such as lecturing large classes, integrating cutting-edge technology, and mentoring graduate students^33^. Using latent-class analysis with 122 German faculty members, a recent study identified four attitudinal profiles; the *optimistic* profile combined the highest AI self-efficacy with the most frequent use of AI-based assessment and feedback, underscoring the motivational role of self-efficacy in university contexts^3^. Furthermore, they typically do not participate in the same curriculum or professional development programs as schoolteachers. Consequently, their concerns about AI may differ in both content and intensity. Existing studies of higher education teachers indicate this uniqueness^34^. For instance, while many higher education teachers appreciate benefits of AI, such as promoting educational equity, a deficiency in AI literacy among both teachers and students is simultaneously reported as a major barrier^35^. Furthermore, distinct profiles of faculty attitudes toward AI, ranging from optimistic to critical, have been identified, with AI self-efficacy strongly predicting AI usage for the optimistic teachers^36^. These findings imply that university teachers not only recognize the promises of AI but also feel they require more support to gain confidence. However, beyond this study, systematic research on AI self-efficacy and AI anxiety in the higher education context, and particularly among Chinese university teachers remains scarce^37^.

### Factors affecting artificial intelligence self-efficacy

Researchers define AI self-efficacy as a multidimensional belief structure that helps explain why some teachers willingly explore AI while others hesitate. Building on recent scale validation work in higher education, this study adopts four widely recognized sub-dimensions of AI self-efficacy: Assistance, Anthropomorphic Interaction, Comfort with AI, and Technological Skills^38,26^. Distinct sources of confidence are captured by each dimension. Collectively, they indicate the readiness of university teachers to incorporate AI into teaching and research.

A foundational dimension in understanding AI self-efficacy is assistance, reflecting teachers’ belief in artificial intelligence’s capacity to reliably augment academic work, from assessment creation to literature synthesis. This perception of AI as a capable assistant appears to significantly lower adoption thresholds, evidenced by a study where educators engaged with generative AI tools by testing how well these AI systems could complete academic assessment tasks and by utilizing them for administrative support^29^. Teachers who highly value the supportive capabilities of AI tend to reallocate saved time to higher order pedagogical tasks, thereby amplifying instructional impact^39^. This assistance effect is often amplified by contextual factors, as teachers who both trust AI support functions and perceive strong institutional backing are significantly more likely to embed AI throughout their teaching^8^.

A second driver of AI self-efficacy is anthropomorphic interaction, reflecting how naturally instructors can converse with chatbots or avatars exhibiting humanlike cues such as turn taking, empathetic wording, or subtle vocal inflection. Social presence theory suggests that as a digital agent signals greater warmth and responsiveness, users more readily transfer interpersonal norms from human dialogue to human AI exchange. Research confirms that even minor anthropomorphic cues are sufficient to trigger users’ sense of engagement, reducing perceived effort and boosting future use intentions^40,41^. Importantly, when teachers view AI as a collaborative partner rather than an obscure tool, their sense of collaboration and willingness to innovate are enhanced^42^. Overall, the evidence suggests that humanization of AI interfaces mitigates psychological distance and initiates a positive feedback loop where successful conversational experiences reinforce confidence and promote deeper adoption.

A further key factor of AI self-efficacy is comfort with AI, an affective state characterized by low tension and high emotional ease whenever AI tools are invoked. Comfort operates as an antecedent that shapes both the depth and persistence of experimentation rather than merely being a passive consequence of successful interactions. Empirical support for this mechanism shows that baseline AI self-efficacy, an indicator of comfort, significantly predicts the subsequent integration of generative AI in teaching work even after controlling perceived usefulness and contextual job variables^39^. Comfort with technology also increases notably when teachers have dedicated time for safe exploration and receive encouragement from peers and administrators, with this heightened comfort in turn positively predicting their intention to adopt generative AI^43^. Collectively, these findings position comfort with AI as a catalytic emotion that converts tentative interest into sustained engagement with AI-enhanced pedagogy.

The final key sub-dimension of AI self-efficacy is technological skills, which evaluates teachers’ self-perceived ability to operate, configure, and troubleshoot AI applications. Mastery experiences are central to this dimension, as each successful instance of refining a prompt or interpreting an algorithmic output creates a feedback loop affirming competence and inviting further experimentation. Crucially, these skills extend beyond basic information and communication technology literacy, encompassing the capacity to refine prompt engineering, interpret al.gorithmic outputs, and apply ethical safeguards. Technological self-efficacy, which reflects teachers’ confidence in these areas, is a powerful predictor of behavior, explaining a significant portion of the variance in actual generative AI tool use beyond demographic factors^38^. Importantly, these skills are teachable, with evidence showing that even compact case-based professional learning on topics like prompt engineering and bias detection can produce large gains in AI related self-efficacy^44^. Complementary qualitative research further indicates that teachers with high AI self-efficacy are more inclined to actively customize AI tools to match disciplinary conventions, reporting stronger student engagement as a result^3^. Therefore, the accumulated evidence strongly suggests that enhancing technological skills is essential for the sustained integration of AI. Without this crucial skill set, even favorable views of AI, such as considering it helpful or user friendly, typically fail to materialize as consistent classroom practice.

In summary, these four dimensions collectively paint a comprehensive picture of Artificial Intelligence Self-Efficacy. It is not a monolithic belief but a multifaceted construct where confidence in the practical assistance of AI, the ease of human-like interaction, a general feeling of comfort, and concrete technological skills all play crucial roles. Studies strongly suggest that these factors are interdependent. For example, strong technical skills can foster greater comfort, while a positive interaction experience can enhance the perception of AI as a helpful assistant. Efforts to boost teachers’ AI self-efficacy must therefore be holistic, addressing not just technical training but also the psychological and affective aspects of technology adoption.

### Factors affecting artificial intelligence anxiety

While self-efficacy captures a teacher’s confidence, Artificial Intelligence Anxiety represents the other side of the coin: the apprehension teachers experience regarding the educational and professional implications of AI. This anxiety is not a single feeling but a complex emotional response. Drawing on established frameworks and recent empirical work, this study analyzes AIAS through four complementary facets, learning anxiety, job replacement anxiety, sociotechnical blindness, and configuration anxiety. Each facet reveals a unique source of unease that can hinder AI adoption.

Among the facets of AI anxiety, AI Learning Anxiety often represents the primary challenge for teachers, revolving around uncertainty about their capacity to keep pace with rapidly evolving AI tools. Cognitive appraisal theory suggests that when instructors judge the learning demands of a new system as exceeding their available resources, they experience stress and protective avoidance. This dynamic is confirmed by survey data showing perceived platform complexity is positively associated with AI learning anxiety and through that pathway can halve pre-service teachers’ likelihood of enrolling in optional AI workshops^45^. However, this feeling of being overwhelmed is highly contingent on instructional design. A case-based narrative onboarding module can reduce cognitive load and learning anxiety while increasing exploratory use of generative tools^44^. These findings indicate that teachers are not inherently technophobic, and that professional development which sequences skill acquisition, highlights relevance, and provides immediate positive feedback appears most effective in transforming apprehension into confident engagement.

In contrast to learning anxiety rooted in competence evaluation, job replacement anxiety stems from anticipated threats to professional roles and identity. Research shows that job replacement anxiety can suppress engagement in professional development activities^1,19^. However, the relationship is not always linear. A four-wave longitudinal study revealed an inverted U-shaped relationship, where moderate anxiety can motivate upskilling, but anxiety beyond a critical threshold precipitates a sharp decline in such intentions^46^. Importantly, narrative framing of AI’s role can serve as a mitigator. When AI was presented as an assistant for routine tasks rather than a replacement, faculty members’ reported anxiety fell and interest in supportive applications increased^3^. These studies characterize job replacement anxiety as a dynamic factor whose impact on professional adaptation is shaped by its perceived intensity and the narrative context of AI’s integration.

Another significant dimension of AI anxiety stems from sociotechnical blindness, the unease that arises when AI systems function as *black boxes* with opaque decision-making processes. As argued by Pasquale, such algorithmic opacity fundamentally undermines accountability and trust^47^. In educational contexts, teachers’ professional agency and sense of responsibility make this issue particularly acute^48^. When an AI tool assigns a grade without offering a clear justification, teachers are left unable to validate the decision, leading to a perceived loss of professional control that is a critical source of anxiety. However, this anxiety can be addressed through increasing transparency and participation. Providing instructors with auditable dashboards reduces their anxiety while increasing trust^49^, and data literacy training helps teachers develop a more critical and confident stance^50^. Qualitative research further shows that when teachers actively participate in AI tool development, their perspective shifts from seeing AI as a threat to viewing it as a co-constructed partner^51^.

AI configuration anxiety reflects the discomfort teachers feel when an intelligent system takes on a physical or hyper-realistic avatar form that appears uncannily human. This unease stems from the blurred boundary between machine and person, which undermines a user’s certainty about agency and responsibility, often leading to behavioral avoidance as a coping strategy. A controlled experiment confirms this, showing that hyper-realistic animated avatars increased feelings of creepiness, which in turn reduced trust and the willingness to reuse the AI assistant compared to a simple chat interface^52^. However, design flexibility can cushion this effect. For example, a workplace learning study found that allowing participants to customize features such as visual realism or voice intonation significantly reduced their configuration anxiety without compromising learning outcomes^53^. These findings suggest that practical strategies such as customizable interfaces and gradual exposure can help transform initial discomfort into acceptance, effectively addressing AI configuration anxiety.

In conclusion, these four dimensions of anxiety reveal that teachers’ apprehension toward AI is complex and multifaceted. It is not simply a fear of technology but a nuanced response to perceived threats to their competence, professional identity, autonomy, and even their sensory comfort. Evidence indicates that these anxieties are not static. They can be intensified by opaque systems and poor design, but can also be mitigated through thoughtful interventions such as targeted training, transparent interfaces, and collaborative design processes. This suggests that addressing AI anxiety requires a deep understanding of the human factors at play, in addition to technical solutions.

### Relationship between artificial intelligence self-efficacy and artificial intelligence anxiety in higher education

Understanding the interplay between these two psychological constructs is crucial, as their combined effect significantly shapes how university teachers engage with AI technologies. Foundational theories, such as Bandura’s^54^ work on self-efficacy, suggest an inverse relationship where greater confidence in one’s abilities typically corresponds with reduced anxiety regarding a specific task. This dynamic is particularly pertinent in the context of AI, where its perceived complexity and rapid evolution can simultaneously challenge teachers’ sense of competence and evoke apprehension.

Empirical evidence largely supports this relationship in educational settings. Studies consistently indicate that higher AI self-efficacy often correlates with lower anxiety, meaning teachers who feel more capable of understanding and using AI are less likely to be burdened by its perceived threats^55,56^. This interaction has direct practical implications for higher education institutions. Enhancing AI self-efficacy, for example, through comprehensive training that builds practical skills and conceptual understanding, may also be associated with lower levels of AI anxiety^56^. Furthermore, professional development addressing both technical proficiency and common AI-related concerns can foster positive attitudes towards these emerging technologies^1^.

However, the connection between AI self-efficacy and AI anxiety is not invariably straightforward and can be moderated by other factors. For example, even with increased AI self-efficacy, teachers who hold fundamental reservations about the pedagogical value of AI may still exhibit reluctance towards its adoption^3^. Furthermore, research highlights a more complex interaction where anxiety, under certain conditions, might even spur action. Notably, a study involving undergraduates found that AI anxiety increased motivation to learn about AI, but critically, only when self-efficacy was also high, suggesting apprehension can motivate learning if individuals feel capable of mastering the new technology^55^. This implies that persistent high levels of AI anxiety, particularly when coupled with low AI self-efficacy, can actively undermine efforts to build confidence by leading to avoidance of necessary learning experiences.

Therefore, AI self-efficacy and AI anxiety are not isolated constructs but are dynamically interconnected, jointly influencing the landscape of AI adoption in higher education. Teachers possessing strong AI self-efficacy across its various dimensions are more likely to perceive AI as a valuable tool and engage with it proactively. Conversely, those experiencing high levels of AI anxiety regarding learning, job security, or broader implications may exhibit greater resistance. For institutions, notably in fast-developing settings like Chinese universities, comprehending the interplay of AI self-efficacy and AI anxiety is essential. This knowledge informs the creation of effective support systems, professional growth opportunities, and guiding policies. Such initiatives help faculty confidently and responsibly integrate AI, ultimately boosting teaching quality and student learning outcomes.

Although the general interplay in higher education between AI self-efficacy and AI anxiety is recognized, the specific manifestation and interrelation of these constructs within the Chinese university teachers present a compelling area for further research. Understanding how these factors vary across diverse gender, age, and major within this setting is also essential. Accordingly, this study is guided by the following research questions:

1. What is the level of participants’ artificial intelligence self-efficacy and artificial intelligence anxiety?
2. Do participants’ artificial intelligence self-efficacy and artificial intelligence anxiety differ by gender?
3. Is age significantly associated with participants’ artificial intelligence self-efficacy and artificial intelligence anxiety?
4. Do participants’ artificial intelligence self-efficacy and artificial intelligence anxiety differ according to their major category?
5. Is there a significant relationship(s) between participants’ artificial intelligence self-efficacy and artificial intelligence anxiety?

## Method

This study quantitatively investigates the relationship between university teachers’ artificial intelligence self-efficacy and artificial intelligence anxiety.

### Participants

A total of 350 full-time teachers (197 female, 153 male, mean age = 41.90, SD = 8.42) from a public normal university in Sichuan Province, China, participated in this study. The sample size was determined using Krejcie and Morgan’s^57^ formula based on a total faculty population of 1,381, yielding a minimum requirement of 302 participants. To accommodate potential incomplete responses, 370 questionnaires were distributed with 350 valid questionnaires returned, representing an effective response rate of 94.6%.

Stratified random sampling was employed to ensure proportional representation across majors. Strata were defined according to the university’s official faculty classification into four broad disciplinary categories: (a) Social Sciences and Humanities (*n* = 175, 50.0%), (b) Arts (*n* = 46, 13.1%), (c) Natural Sciences with Computer Science (*n* = 18, 5.2%), and (d) Natural Sciences without Computer Science (*n* = 111, 31.7%). Within each stratum, participants were randomly selected in proportion to the stratum’s share of the total faculty population. Table 1 presents the full demographic profile.

**Table 1:** Demographic information of the participants (*N* = 350).

Variable	Categories	*N*	Frequency (%)
Gender	Female	197	56.3
	Male	153	43.7
Age	Under 32	59	16.8
	33–41	127	36.4
	42–50	105	30.0
	51–59	59	16.8
Major category	Social Sciences and Humanities	175	50.0
	Arts	46	13.1
	Natural Sciences (Computer Science)	18	5.2
	Natural Sciences (Non-Computer Science)	111	31.7

### Instruments

#### Artificial intelligence self-efficacy scale (AISE)

The AI Self-Efficacy Scale (AISE), developed by Wang and Chuang^26^, was adopted for this study. For this study, the scale was translated into Chinese without modifying the original items. The scale comprised 22 items rated on a 7-point Likert scale, assessing the four sub-dimensions of assistance (AS), anthropomorphic interaction (AI), comfort with AI (CF), and technological skills (TS). In the original validation study, the Cronbach’s alpha values demonstrated excellent internal consistency for the overall scale (α = 0.958) and for each of the four sub-dimensions: AS (α = 0.942), AI (α = 0.970), CF (α = 0.963), and TS (α = 0.869), which demonstrates high internal consistency^58^. Sample items include: I find that AI technologies/products are helpful for learning. When interacting with AI technologies/products, I feel very calm.

A pilot study (*n* = 120) showed acceptable to good internal consistency for the total AISE score (Cronbach’s α = 0.83) and robust reliabilities for the four subscales (AS α = 0.87; AI α = 0.78; CF α = 0.85; TS α = 0.82). Sampling adequacy was adequate (KMO = 0.79), and Bartlett’s test was significant (χ² = 1053.23, df = 231, *p* < .01), supporting factorability. Confirmatory factor analysis extracted the expected four‑factor solution; after rotation these factors accounted for about 58.7% of the total variance, and targeted items loaded strongly on their intended factors.

#### Artificial intelligence anxiety scale (AIAS)

The AI Anxiety Scale (AIAS), developed by Wang and Wang^22^, was adopted for this study. For this study, the scale was translated into Chinese without any modification to the original items. The scale comprised 21 items rated on a 7-point Likert scale, assessing the four sub-dimensions of learning, job replacement (JR), sociotechnical blindness (SB), and AI configuration (AIC). The original study reported Cronbach’s alpha of 0.964 for the overall scale. The reliability for the individual sub-scales was also high: L (α = 0.974), JR (α = 0.917), SB (α = 0.917), and AIC (α = 0.961), indicating excellent internal consistency^58^. Sample items include: Learning to use AI techniques/products makes me anxious and I am afraid that AI techniques/products will replace someone’s job.

In the same pilot sample, AIAS demonstrated good internal consistency for the total scale (Cronbach’s α = 0.88) and strong reliability across subscales (L α = 0.89; JR α = 0.85; SB α = 0.86; AIC α = 0.79). Sampling adequacy was good (KMO = 0.84), and Bartlett’s test was significant (χ² = 1164.44, df = 210, *p* < .01), indicating suitability for factor analysis. A confirmatory four‑factor solution consistent with the hypothesized structure emerged; the rotated factors jointly explained about 62.5% of the variance, and items showed substantive loadings on their intended dimensions.

### Demographic information

A demographic form of 3 questions was used to obtain demographic information about age, gender, and major category of the participants.

### Data collection and data analysis

This study received ethical approval from the university’s research ethics committee. Participant recruitment and data collection were conducted from September to November 2025, after ethical approval had been granted. Prior to data collection, written informed consent was obtained from all participants, who were teachers from a normal university in Sichuan, China, and permission to use and translate the AISE and AIAS was also obtained from the original authors. Participants were thoroughly briefed on the research objectives and assured that the questionnaire would take approximately 20 min to complete. They were explicitly informed that their participation was voluntary, and they could withdraw from the survey at their convenience without providing any reason or facing any repercussions. The confidentiality of all collected data was guaranteed, and the researcher’s contact information was provided for any further clarifications.

Data was analyzed using SPSS Statistics for Windows, Version 29.0 (IBM Corp., Armonk, NY, USA). Descriptive statistics, including frequencies, percentages, means, and standard deviations, were used to report the demographic characteristics of the participants and their scores on the AI self-efficacy and AI anxiety scales. One-sample t-tests were conducted to compare the mean AI self-efficacy and AI anxiety scores, as well as their subscale scores, against the neutral midpoint of 4 on the 7-point Likert scale. Pearson correlations were computed to examine associations among age, AI self-efficacy, AI anxiety, and their subdimensions. Inferential statistics were employed to examine group differences. Specifically, independent samples t-tests and one-way analysis of variance (ANOVA) were used to compare the mean scores on the scales across different demographic groups. Assumptions of normality and homogeneity of variances were examined prior to conducting t-tests and ANOVA. Cohen’s d was computed for all t-tests to provide standardized effect size estimates, interpreted using Cohen’s (1988) conventional benchmarks of small (0.20), medium (0.50), and large (0.80). Partial eta-squared values were reported for all ANOVA. For any ANOVA that yielded a significant F-test, a post-hoc multiple comparison test using the Bonferroni test was employed to identify specific group differences. Additionally, hierarchical multiple regression was conducted to examine the unique predictive contribution of AI self-efficacy to AI anxiety after controlling for demographic variables. Prior to regression, assumptions of linearity, normality of residuals, homoscedasticity, independence of errors, and multicollinearity (tolerance > 0.20, VIF < 5) were assessed. The level of statistical significance for all inferential tests was set at *p* < .05.

## Results

### Descriptive statistics and preliminary analyses

Descriptive statistics for the two scales and their respective sub-dimensions are presented in Table 2. The skewness and kurtosis values for all variables fell within the acceptable range of -2 to + 2, indicating that the data conform to a normal distribution. Visual inspection of Q-Q plots further confirmed approximate normality for all continuous variables. Furthermore, Cronbach’s alpha values for both scales and dimensions ranged from 0.79 to 0.88, demonstrating good to excellent internal consistency and reliability for the measurement instruments used in this study^58^.

**Table 2:** Descriptive information.

	Min	Max	M	SD	Skewness	Kurtosis	Cronbach’s α
AISE	62	149	98.57	16.7	0.51	0.23	0.82
AS	12	49	31.69	7.74	0.01	-0.33	0.79
AI	6	35	21.75	6.28	-0.02	-0.57	0.81
CF	9	42	26.68	7.58	0.03	-0.69	0.81
TS	5	28	18.45	5.28	-0.19	-0.68	0.81
AIAS	35	131	91.29	17.92	-0.55	0.14	0.85
L	11	54	35.69	8.59	-0.51	-0.10	0.79
JR	8	42	25.49	7.33	-0.12	-0.77	0.84
SB	4	28	18.00	6.15	-0.32	-0.78	0.88
AIC	3	21	12.11	4.21	-0.09	-0.56	0.79

M=Mean; SD=Standard Deviation. AISE = Artificial Intelligence Self-Efficacy Scale; AS = Assistance; AI = Anthropomorphic Interaction; CF = Comfort with AI; TS = Technological Skills. AIAS = Artificial Intelligence Anxiety Scale; L = Learning; JR = Job Replacement; SB = Sociotechnical Blindness; AIC = AI Configuration.

One-sample t-test results for the AISE and its sub-factors are shown in Table 3. Since 4 represents the neutral point on the 7-point Likert scale (1 = strongly disagree, 2 = disagree, 3 = somewhat disagree, 4 = neither agree nor disagree, 5 = somewhat agree, 6 = agree, 7 = strongly agree), it was selected as the test value to determine whether participants’ AI self-efficacy differed significantly from neutrality. The mean scores for AISE and all its sub-factors were found to be significantly higher than 4 (*p* < .01). This suggests that the participants generally hold positive and confident self-efficacy towards their ability to understand and interact with AI.

**Table 3:** One-sample t-test for AISE.

	M	SD	MD	t (df = 349)
AISE_m	4.48	0.76	0.48	11.80*
AS_m	4.53	1.11	0.53	8.91*
AI_m	4.35	1.26	0.35	5.21*
CF_m	4.45	1.26	0.45	6.61*
TS_m	4.61	1.32	0.61	8.69*

M=Mean; SD=Standard Deviation; MD=Mean Difference; df=Degrees of Freedom. AISE=Artificial Intelligence Self-Efficacy Scale; AS=Assistance; AI=Anthropomorphic Interaction; CF=Comfort with AI; TS=Technological Skills. Test value = 4. *p < .01.

Similarly, one-sample t-test results for the AIAS and its subfactors are presented in Table 4. The AIAS is a 7-point Likert scale (1 = strongly disagree, 2 = disagree, 3 = somewhat disagree, 4 = neither agree nor disagree, 5 = somewhat agree, 6 = agree, 7 = strongly agree), where 4 represents a neutral attitude and was therefore selected as the test value to determine if participants’ anxiety scores significantly deviated from this point. The results show that the mean score for the total AIAS (M = 4.35, SD = 0.85) was significantly higher than the neutral value (t = 7.62, *p* < .01), indicating that participants experienced notable anxiety regarding AI. This anxiety was also reflected across several subfactors. The mean scores for learning (M = 4.46, SD = 1.07), job replacement (M = 4.25, SD = 1.22), and sociotechnical blindness (M = 4.50, SD = 1.54) were all significantly higher than the neutral point (*p* < .01 for all), suggesting participants are particularly anxious about learning to use AI, the potential for job displacement, and its broader societal consequences. In contrast, the mean score for AI configuration anxiety (M = 4.04, SD = 1.40) showed no significant difference from the neutral value of 4 (t = 0.50, *p* > .05). This result indicates that there is insufficient evidence to conclude that participants hold specific anxiety about the technical aspects of configuring or setting up AI systems.

**Table 4:** One-sample t-test for AIAS.

	M	SD	MD	t (df = 349)
AIAS_m	4.35	0.85	0.35	7.62*
L_m	4.46	1.07	0.46	8.03*
JR_m	4.25	1.22	0.25	3.82*
SB_m	4.50	1.54	0.50	6.08*
AIC_m	4.04	1.40	0.04	0.50

M=Mean; SD=Standard Deviation; MD=Mean Difference; df=Degrees of Freedom. AIAS=Artificial Intelligence Anxiety Scale; L=Learning; JR = Job Replacement; SB=Sociotechnical Blindness; AIC=Artificial Intelligence Configuration. Test value = 4. *p < .01.

### Relationships between AI self-efficacy and AI anxiety

Table 5 displays the correlations between the variables. Age showed a significant negative correlation with the total AIAS score (*r* = -0.16, *p* < 0.01) and was particularly negatively correlated with the anxiety subfactor of L (*r* = -.28, *p* < .01), indicating that as participants get older, their anxiety about learning new AI systems tends to decrease. A key finding is the significant, strong negative correlation between the total AIAS and AISE scores (*r* = -.59, *p* < .01), confirming that higher levels of AI anxiety are strongly associated with lower levels of AI self-efficacy. A more detailed test of cross-scale correlations reveals that this inverse relationship is remarkably consistent and pervasive across nearly all subfactors. Specifically, all four AIAS subfactors consistently showed significant negative correlations with all four AISE subfactors, suggesting that a participants’ anxiety is not an isolated fear but is systematically linked to a broader lack of confidence. For instance, a participants’ anxiety about job replacement is not only associated with lower confidence in their technical skills (TS, *r* = -0.21, *p* < .01) but is also strongly tied to lower self-efficacy in getting assistance (AS, *r* = -0.26, *p* < .01) and feeling comfortable with AI systems (CF, *r* = -0.30, *p* < 0.01). These results indicate that job replacement was negatively associated with multiple dimensions of AI self-efficacy, including technological skills, assistance, and comfort with AI. The results indicate that participants’ self-efficacy, particularly their confidence in application and comfort (AS, AI, CF), is closely and inversely associated with the entire spectrum of AI anxiety, although the cross-sectional design precludes directional or causal inference.

**Table 5:** Correlations between AI anxiety and AI self-efficacy.

	Age	L	JR	SB	AIC	AIAS	AS	AI	CF	TS	AISE
L	-0.28**	1									
JR	0.10	0.29**	1								
SB	-0.20**	0.36**	0.34**	1							
AIC	-0.06	0.16**	0.23**	0.12*	1						
AIAS	-0.16**	0.64**	0.69**	0.73**	0.59**	1					
AS	0.16**	-0.31**	-0.26**	-0.31**	-0.22**	-0.42**	1				
AI	0.12*	-0.21**	-0.24**	-0.28**	-0.17**	-0.34**	0.22**	1			
CF	0.11*	-0.31**	-0.30**	-0.34**	-0.28**	-0.46**	0.20**	0.19**	1		
TS	-0.24**	-0.11*	-0.21**	-0.12*	-0.22**	-0.25**	0.15**	0.14**	0.14**	1	
AISE	0.05	-0.38**	-0.41**	-0.42**	-0.36**	-0.59**	0.60**	0.63**	0.63**	0.61**	1

L=Learning; JR = Job Replacement; SB=Sociotechnical Blindness; AIC = AI Configuration; AIAS=Artificial Intelligence Anxiety. AS=Assistance; AI=Anthropomorphic Interaction; CF=Comfort with AI; TS=Technological Skills; AISE = AI Self-Efficacy Scale.

**. Correlation is significant at the 0.01 level (2-tailed).

*. Correlation is significant at the 0.05 level (2-tailed).

To further examine the unique contribution of AI self-efficacy to AI anxiety beyond demographic factors, a two-step hierarchical multiple regression was conducted with mean AIAS score as the dependent variable (Table 6). In Step 1, demographic control variables were entered, including gender (coded 1 = female, 2 = male), age (continuous), and major (three dummy variables with Social Sciences and Humanities as the reference category). In Step 2, mean AISE score was added as a predictor.

**Table 6:** Hierarchical multiple regression predicting AI anxiety.

Predictor	Model 1 B	SE	β	*p*	Model 2 B	SE	β	*p*
Constant	6.272	0.160	-	< 0.001	6.690	0.246	-	< 0.001
Gender	-0.663	0.060	− 0.386	< 0.001	-0.634	0.061	− 0.369	< 0.001
Age	-0.014	0.004	− 0.136	< 0.001	-0.013	0.004	− 0.124	< 0.001
Arts	-0.346	0.090	− 0.137	< 0.001	-0.301	0.091	− 0.119	0.001
Natural sciences (non-CS)	-0.789	0.067	− 0.431	< 0.001	-0.688	0.080	− 0.376	< 0.001
Computer science	-2.093	0.134	− 0.542	< 0.001	-1.825	0.179	− 0.473	< 0.001
AISE mean	-	-	-	-	-0.125	0.056	− 0.112	0.026

AISE = AI Self-Efficacy; B = unstandardized coefficient; SE = standard error; β = standardized coefficient.

Prior to analysis, regression assumptions were verified. Tolerance values for all predictors in the final model ranged from 0.452 to 0.89 (all > 0.20), and VIF values ranged from 1.12 to 2.21 (all < 5), indicating no multicollinearity. The Durbin-Watson statistic was 2.005, suggesting independent residuals. Visual inspection of residual plots confirmed approximate homoscedasticity.

Step 1 (demographic variables alone) accounted for a substantial proportion of variance in AI anxiety (R² = 0.604, adjusted R² = 0.599, F(5, 344) = 105.050, *p* < .001). Among the demographic predictors, major emerged as the strongest factor. Specifically, compared to the Social Sciences and Humanities reference group, computer science faculty reported substantially lower anxiety (B = -2.093, β = − 0.542, *p* < .001), followed by non-computer-science natural sciences (B = -0.789, β = − 0.431, *p* < .001) and arts (B = -0.346, β = − 0.137, *p* < .001).Gender was also a significant predictor of anxiety (B = -0.663, β = − 0.386, *p* < .001); given the coding used in this study, this negative coefficient indicates that male teachers reported lower anxiety than female teachers. Older age was associated with slightly lower anxiety (B = -0.014, β = − 0.136, *p* < .001).

Step 2, which added mean AISE score, produced a statistically significant increment in explained variance (ΔR² = 0.006, F change(1, 343) = 4.990, *p* = .026), with the final model explaining R² = 0.610 of the variance (adjusted R² = 0.603, F(6, 343) = 89.388, *p* < .001). In the final model, AISE mean score emerged as a significant negative predictor of AIAS (B = -0.125, β = − 0.112, *p* = .026), indicating that higher overall AI self-efficacy was associated with lower AI anxiety even after controlling for gender, age, and major. Notably, gender (β = − 0.369, *p* < .001) and major (computer science, β = − 0.473, *p* < .001) retained their strong predictive effects in the final model, suggesting that demographic and disciplinary context account for a large portion of anxiety variance independent of AI self-efficacy.

### Group differences by gender and major

To further interpret the demographic and disciplinary effects observed in the regression model, group comparison analyses were conducted by gender and major category.

The results of the independent t-tests examining gender differences are presented in Table 7. A significant gender difference was found in the total AIAS score (t = 9.87, d = 1.08, *p* < .05), indicating a large effect, with females reporting significantly higher overall anxiety towards AI compared to males. This pattern of heightened anxiety among females was consistent across the subfactors of L (t = 6.42, d = 0.70, *p* < .05), JR (t = 6.88, d = 0.74, *p* < .05), and SB (t = 12.19, d = 1.33, *p* < .05), revealing that the gender gap is most pronounced when considering the abstract and future-oriented challenges of adapting to AI, such as learning new systems, the threat of job displacement, and potential societal disruption. The effect sizes for SB (d = 1.33) and the total AIAS (d = 1.08) were particularly large, indicating substantial and practically meaningful gender differences in these dimensions. Conversely, a significant gender difference was also observed in the total AISE score (t = -4.65, d = -0.50, *p* < .05), where males demonstrated a significantly higher self-efficacy. This confidence was specifically reflected in the sub-dimensions of AS (t = -3.57, d = -0.38, *p* < .05), AI (t = -2.77, d = -0.30, *p* < .05), and CF (t = -4.72, d = -0.51, *p* < .05). Taken together, these results show that male teachers reported higher self-efficacy on several dimensions and lower anxiety on the overall AIAS and selected anxiety subfactors. Notably, this pattern of divergence has clear boundaries. The analysis revealed no significant gender gap for either AIC anxiety (*p* = .188, d = -0.14) or for TS self-efficacy (*p* = .563, d = 0.06). Both effect sizes were negligible, suggesting that when both genders perceive their foundational technological skills as equivalent, they also share a similar level of anxiety about the specific, task-oriented challenge of AI configuration. No significant gender differences were found for AIC anxiety or TS self-efficacy, and both effect sizes were negligible.

**Table 7:** T-Tests by Gender.

	Gender	df	M	SD	MD	t	*p*	Cohen’s d
AIAS	Female	309.08	98.75	15.08	17.04	9.87*	0.00	1.08
	Male		81.71	16.72				
L	Female	305.96	38.18	7.68	5.71	6.42*	0.00	0.70
	Male		32.48	8.66				
JR	Female	327.69	27.73	6.91	5.11	6.88*	0.00	0.74
	Male		22.62	6.87				
SB	Female	313.27	20.98	4.96	6.83	12.19*	0.00	1.33
	Male		14.16	5.38				
AIC	Female	315.67	11.85	4.08	– 0.60	– 1.32	0.188	– 0.14
	Male		12.45	4.36				
AISE	Female	325.81	95.00	16.24	– 8.17	– 4.65*	0.00	– 0.50
	Male		103.17	16.35				
AS	Female	333.14	30.42	7.76	– 2.92	– 3.57*	0.00	– 0.38
	Male		33.33	7.43				
AI	Female	323.59	20.93	6.17	– 1.86	– 2.77*	0.006	– 0.30
	Male		22.80	6.30				
CF	Female	333.36	25.05	7.50	– 3.72	– 4.72*	0.00	– 0.51
	Male		28.77	7.17				
TS	Female	320.83	18.60	5.20	0.33	0.58	0.563	0.06
	Male		18.27	5.40				

df=Degrees of Freedom; M=Mean; SD=Standard Deviation; MD=Mean Difference. AISE Scale: AS=Assistance; AI=Anthropomorphic Interaction; CF=Comfort with AI; TS=Technological Skills. AIAS Scale: L=Learning; JR = Job Replacement; SB=Sociotechnical Blindness; AIC=Artificial Intelligence Configuration. *p < .05.

Major differences were also examined. Firstly, the assumption of homogeneity of variances was checked. As shown in Table 8, Levene’s test was not significant for any of the tested variables (*p* > .05), so that equal variances were assumed. The results of ANOVA are presented in Table 9. The analysis revealed that there were statistically significant differences among the academic major categories across both total scales and all tested sub-dimensions. Specifically, evidence of significant differences was found for the total AIAS score (F = 82.89, *p* < .001, ηp² = 0.42), the total AISE score (F = 118.38, *p* < .001, ηp² = 0.51), and the sub-dimensions of JR (F = 40.77, *p* < .001, ηp² = 0.26), AIC (F = 22.78, *p* < .001, ηp² = 0.165), and AI (F = 29.86, *p* < .001, ηp² = 0.20). According to Cohen’s (1988) guidelines, these ηp² values indicate medium to large effect sizes, with the largest effect observed for AISE and the smallest for AIC.

**Table 8:** Test of homogeneity of variances.

JR	Levene Statistic	df1	df2	*p*
	1.12	3	346	0.343
AIC	0.18	3	346	0.907
AIAS	0.88	3	346	0.450
AI	0.69	3	346	0.561
AISE	2.02	3	346	0.111

df=Degrees of Freedom. JR = Job Replacement; AIC = AI Configuration; AIAS=Artificial Intelligence Anxiety Scale; AI=Anthropomorphic Interaction; AISE = AI Self-Efficacy.

**Table 9:** ANOVA.

		SS	df	MS	F	*p*	ηp²	Bonferroni Post-Hoc
JR	Between groups	4907.75	3	1635.92	40.77	< 0.001	0.26	HS < ART< NSN < NSC
	Within Groups	13881.75	346	40.12				
	Total	18789.50	349					
AI	Between Groups	2833.49	3	944.50	29.86	< 0.001	0.20	HS < ART< NSN < NSC
	Within Groups	10944.38	346	31.63				
	Total	13777.87	349					
AIC	Between Groups	1020.61	3	340.20	22.78	< 0.001	0.165	NSC < NSN< ART < HS
	Within Groups	5168.04	346	14.94				
	Total	6188.65	349					
AIAS	Between Groups	46861.90	3	15620.63	82.89	< 0.001	0.42	NSC < NSN< ART < HS
	Within Groups	65205.20	346	188.45				
	Total	112067.10	349					
AISE	Between Groups	49655.22	3	16551.74	118.38	< 0.001	0.51	HS < ART< NSN < NSC
	Within Groups	48378.49	346	139.82				
	Total	98033.71	349					

SS = Sum of Squares; df=Degrees of Freedom; MS=Mean Square. JR = Job Replacement; AI=Anthropomorphic Interaction; AIC = AI Configuration; AIAS=Artificial Intelligence Anxiety Scale; AISE = AI Self-Efficacy.

Bonferroni post-hoc comparisons revealed a consistent pattern across most dimensions. Participants from the computer science group generally scored highest on self-efficacy-related measures (e.g., AISE, AI) and lowest on anxiety-related measures (e.g., AIAS, JR), while participants from the humanities and social sciences group exhibited the opposite pattern. Arts and non-computer-science natural sciences groups tended to fall between these extremes, with arts often outperforming humanities and social sciences but scoring lower than non-computer-science natural sciences and computer science groups. Overall, the computer science group reported the highest self-efficacy and the lowest anxiety, whereas the humanities and social sciences group showed the opposite pattern.

## Discussion and conclusion

This study investigated the complex relationship between artificial intelligence self-efficacy and anxiety among university teachers in a Chinese higher education setting. The findings reveal a state of what can be termed *confident apprehension*, where teachers simultaneously report moderate-to-high capability in using AI and significant anxiety about its broader implications. This apparent paradox may reflect that teachers distinguish between their confidence in using AI as a practical tool from their deeper anxieties about its transformative impact on their profession and the educational landscape. Their confidence aligns with literature indicating that teachers recognize AI’s practical benefits for tasks like curriculum planning and assessment^4,3^. However, this confidence does not mitigate anxieties about learning, job displacement, and sociotechnical blindness. This reflects a deeper concern about the fundamental shifts AI might bring to their professional roles, a finding consistent with explorations of teacher anxiety related to career and skills^19,18^.

A central finding of this study is the robust and pervasive inverse relationship between AI self-efficacy and AI anxiety (*r* = -.59). This strong negative correlation suggests that self-efficacy is more than a concurrent psychological state. It is closely associated with reduced apprehension, a pattern that is theoretically consistent with Bandura’s (1977) proposition that stronger capability beliefs correspond to lower task-related anxiety. This empirically supports the foundational theory of self-efficacy and aligns with recent AI-related educational studies^55,56^. The detailed cross-scale correlation analysis extends this understanding, showing that confidence in any single facet of AI use, such as practical assistance or comfort, is inversely associated with multiple types of anxiety, ranging from learning difficulties to job replacement fears. This pattern suggests that genuine competence and reduced anxiety are closely intertwined, and that professional development targeting self-efficacy may also help address apprehensions that hinder AI integration, a hypothesis that warrants longitudinal testing.

The hierarchical regression analysis provided additional depth to this understanding. Even after controlling for the strong effects of gender, age, and major, which together explained 60.4% of the variance in AI anxiety, total AI self-efficacy contributed a statistically significant additional 0.57% of explained variance (*p* = .026). While modest in magnitude, this finding confirms that the self-efficacy and anxiety association is not merely an artifact of shared demographic or disciplinary characteristics. This pattern is theoretically consistent with Bandura’s proposition that efficacy beliefs help shape emotional responses to challenging tasks, and it also aligns with recent research showing that stronger AI-related self-efficacy is associated with lower anxiety or more adaptive engagement with AI-related learning demands^55^;^56^. At the same time, the very small increment in explained variance suggests that, in this sample, self-efficacy alone cannot account for the broader structure of AI anxiety. In this respect, the present findings refine more general expectations in the literature that strengthening confidence will automatically lead to lower apprehension. Instead, they suggest that AI self-efficacy operates as one protective factor within a wider constellation of contextual influences. This interpretation is consistent with studies showing that teachers’ AI adoption is shaped not only by internal beliefs but also by institutional support, disciplinary culture, and the broader conditions under which AI is introduced^8,3^. Notably, the dominance of demographic predictors in the regression model indicates that contextual factors, particularly major and gender, were the strongest predictors of AI anxiety in this sample. Accordingly, the regression findings support a more nuanced interpretation of the AISE-AIAS relationship. Higher self-efficacy matters, but its role is embedded within the social and disciplinary realities of university teaching rather than operating in isolation. This has important practical implications, suggesting that institutional interventions should be differentiated by discipline and should attend to gender-related confidence gaps, rather than relying solely on general self-efficacy enhancement programs.

The study also uncovers significant demographic and disciplinary differences in the pattern of results. A pronounced gender gap was observed, with female teachers reporting higher anxiety and lower self-efficacy. The large effect sizes for overall AIAS (d = 1.08) and sociotechnical blindness (d = 1.33) indicate that these are not trivial differences but substantial and practically meaningful disparities. This resonates with research identifying heightened concern among female instructors when adopting new technologies^1^. Critically, this gap vanished when examining foundational technological skills and the associated configuration anxiety. The observed gender disparities may reflect social and institutional influences rather than inherent differences in ability or disposition. Socialization processes, differential access to technology-intensive roles, culturally embedded gender norms regarding technical domains, and unequal representation in STEM-oriented professional development may help explain why female teachers reported lower AI self-efficacy and higher anxiety^1^. The negligible effect sizes for TS self-efficacy (d = 0.06) and AIC anxiety (d = -0.14) are consistent with this possibility, as both genders reported similar levels of foundational technological self-efficacy, whereas the divergence was more evident in broader AI-related confidence and anxiety. Consequently, institutional interventions should target the social and environmental factors that shape confidence rather than assuming deficits in ability.

Furthermore, major emerged as a strong predictor of teachers’ psychological readiness for AI. The emergence of a clear hierarchy points to the possibility of an *internal AI divide* within universities. This hierarchy places computer science faculty at one extreme, with the highest self-efficacy and lowest anxiety, and humanities and social sciences faculty at the other. This finding provides strong empirical weight to studies suggesting that disciplinary cultures frame how technology is perceived and used^33,3^. The large effect size for AISE (ηp² = 0.51) indicates this is not a trivial difference but a substantial disparity in psychological readiness. The particularly high job replacement anxiety among humanities teachers underscores a tangible fear that AI will devalue creative and human-centric disciplines, a concern that institutional training must address directly.

Interestingly, this study found that older teachers reported less anxiety about learning new AI systems. This counter-intuitive result challenges the conventional wisdom that veteran educators are more resistant to technology. A possible explanation may be that extensive pedagogical experience and a secure professional identity may foster greater psychological resilience. Whereas some research highlights that AI can pose a threat to teachers’ professional roles and skills^19,18^, the findings on age in this study suggest an alternative dynamic. It may indicate that more experienced teachers are better able to frame AI as another tool to be incorporated into established practice, rather than as a fundamental threat to their competence. This pattern was especially evident for learning-related anxiety. Taken together, this age-related pattern suggests that anxiety about AI may be shaped not only by exposure to technology but also by how teachers situate AI within a longer professional trajectory.

In conclusion, this study reveals that Chinese university teachers exist in a state of confident apprehension regarding AI. While feeling capable of using technology, they remain anxious about its broader professional and societal implications. This dynamic is significantly shaped by gender and, most powerfully, major. The most critical finding is the strong inverse relationship between self-efficacy and anxiety, suggesting that enhancing teachers’ confidence through targeted, holistic support represents a promising strategy for alleviating their apprehensions and fostering meaningful AI adoption in higher education, a proposition that future intervention studies should empirically verify. At the same time, the regression findings indicate that self-efficacy alone is unlikely to be sufficient unless universities also address the disciplinary and social contexts in which AI-related concerns are formed.

### Implication

The results of this study offer significant implications for institutional policy, theoretical understanding and future research directions. From a practical standpoint, institutions should move beyond uniform approaches to AI training and instead develop differentiated and holistic professional development. Programs for humanities and social science teachers should prioritize building foundational confidence and demystifying AI’s function, framing it as a collaborative tool that enhances rather than replaces humanistic inquiry. In contrast, training for teachers already comfortable with technology can delve into advanced pedagogical applications and ethical considerations. Crucially, all training must address both technical skills to foster efficacy and provide a safe forum to discuss anxiety to alleviate fear. Furthermore, universities should implement gender-sensitive support structures, such as female-led mentorship programs and communities of practice, to address the observed confidence gap and nurture a more inclusive environment for AI exploration.

From a theoretical perspective, the present study provides initial evidence for the reliability and applicability of the AISES^26^ and AIAS^22^ instruments within a Chinese higher education context. The acceptable-to-good internal consistency values obtained across both pilot and main studies offer a preliminary foundation for future cross-cultural validation efforts incorporating confirmatory factor analysis and measurement invariance testing. Furthermore, the observed variations in the self-efficacy and anxiety association across gender and disciplinary subgroups suggest that demographic and contextual factors may moderate this relationship, extending previous work that has treated the association as uniform. However, these potential moderating patterns were identified through subgroup comparisons rather than formal interaction testing, and should therefore be regarded as hypotheses for future research rather than confirmed moderating effects.

### Limitations and future research

This study also has limitations. Findings are based on a sample from a single normal university in Sichuan Province, China, which may restrict generalizability. The institutional culture of a teaching-oriented normal university, together with China’s specific national AI-in-education policies, may have shaped participants’ self-efficacy and anxiety levels in ways that differ from research-intensive universities or institutions in other national policy environments. Future research should therefore aim to replicate this study across diverse institutional types, geographic regions, and policy contexts to establish the boundary conditions of the observed relationships.

Additionally, the computer science subgroup (*n* = 18) was small, reflecting the actual proportion of computer science faculty within this institution. While the large effect sizes associated with disciplinary differences lend confidence to these comparisons, the limited sample size within this subgroup may reduce the precision of point estimates and increase the width of confidence intervals. Future studies should oversample from computer science and related technology disciplines to enable more robust subgroup analyses.

The demographic variables in this study were also limited to gender, age, and major. Variables that prior research has identified as relevant to AI adoption, such as years of teaching experience, prior exposure to AI tools, and baseline digital competence^39,38^, were not collected. Future studies should incorporate these variables to provide a richer and more nuanced account of the factors shaping AI self-efficacy and anxiety among university teachers.

The reliance on self-report data also suggests demand for future studies incorporating objective performance-based assessments. Finally, the cross-sectional design of this research provides a static snapshot. Three research directions are particularly urgent. First, longitudinal panel studies tracking the same cohort of teachers over at least two semesters would clarify the temporal dynamics of the self-efficacy and anxiety relationship and determine whether changes in one construct precede changes in the other. Second, quasi-experimental intervention studies, for example comparing discipline-specific AI training workshops with generic programs, are needed to test whether targeted professional development can simultaneously enhance self-efficacy and reduce anxiety. Third, mixed-methods designs incorporating qualitative interviews could illuminate the mechanisms through which confidence is built or eroded, providing the explanatory depth that cross-sectional surveys alone cannot offer.

## Data Availability

The datasets generated during and/or analyzed during the current study are available from the corresponding author on reasonable request.
